# Localized Patch-Based Fuzzy Active Contours for Image Segmentation

**DOI:** 10.1155/2016/1064692

**Published:** 2016-12-13

**Authors:** Jiangxiong Fang, Hesheng Liu, Huaxiang Liu, Liting Zhang, Jun Liu

**Affiliations:** ^1^Fundamental Science on Radioactive Geology and Exploration Technology Laboratory, East China University of Technology, Nanchang 330013, China; ^2^Key Laboratory of Watershed Ecology and Geographical Environment Monitoring, NASG, Nanchang 330013, China; ^3^School of Geophysics and Measure Control Technology, East China University of Technology, Nanchang 330013, China

## Abstract

This paper presents a novel fuzzy region-based active contour model for image segmentation. By incorporating local patch-energy functional along each pixel of the evolving curve into the fuzziness of the energy, we construct a patch-based energy function without the regurgitation term. Its purpose is not only to make the active contour evolve very stably without the periodical initialization during the evolution but also to reduce the effect of noise. In particular, in order to reject local minimal of the energy functional, we utilize a direct method to calculate the energy alterations instead of solving the Euler-Lagrange equation of the underlying problem. Compared with other fuzzy active contour models, experimental results on synthetic and real images show the advantages of the proposed method in terms of computational efficiency and accuracy.

## 1. Introduction

Image segmentation has been one of the most fundamental and important tasks in image analysis and computer vision. Its purpose is to divide the given image into several regions where the inner pixels are homogeneous with respect to some characteristic. Over the past decades, many approaches have been developed to improve the performance of the image segmentation algorithms. Variational formulation [1–3] has become one of the most effective algorithms for image segmentation because it can minimize an objective function containing terms that embedded description of its regions and boundaries. However, the segmentation procedure is still considered as an essential and difficult process, especially due to the variety and complexity of the images.

Active contour models (ACMs) [2–8] have been proved to be an efficient framework for image segmentation. The existing ACMs can mainly be categorized into two classes: edge-based models and region-based models. The edge-based ACMs [2, 3] utilize image gradient to construct an edge stopping function to stop the contour evolution on the object boundaries. These models are highly dependent on the initial contours and easily suffer from serious boundary leakage problems in the position of weak boundaries. The region-based ACMs [4–8] exploit the statistical image intensity information (e.g., intensity, color, and texture) to ensure that the energy functional achieves minima when the contours reach the object boundary. Chan-Vese (CV) model [5], which is based on Mumford-Shah (M-S) model [4], has become one of the well-known region-based ACMs. This model uses the global intensity difference to guide the contour and has succeeded in detecting the objects of which the boundaries are not necessarily defined by gradient. However, in these models, the periodical reinitialization of the level set function (LSF) causes a lot of computations and some numerical errors.

In order to improve the segmentation performance of the region-based ACMs, many multiphase level set methods [7–10] are proposed to segment images with many objects, which leads to a complicated expression of energy functional and greatly increases the computational complexity. Local region-based ACMs [11–14], which combine the region-based techniques with the benefits of local information, have been proposed to segment images with intensity inhomogeneity. Using the local intensity information, Li et al. [11, 12] proposed an efficient region-based level set method driven by a local binary fitting (LBF) energy and has achieved promising results. But the LBF model needs to perform four convolution operations at each iteration, which greatly increases the computational complexity. Brox and Cremers [13] derive a statistical interpretation of the Mumford-Shah functional on local region statistics. Thanks to the analytical expression of the smooth approximation via Gaussian convolution, the coordinate descent can be replaced by a true gradient descent. A characteristic function defined by the radius parameter [14] is used to extract the local information. Three specific energies in the model are introduced that can be inserted as internal energy measures: uniform modeling, means separation, and histogram separation energy.

Later on, the fuzzy energy-based active contour (FEAC) model is introduced by Krinidis and Chatzis [15] to reject local minima. In the model, a fast optimization algorithm is applied to minimize the fuzzy energy function instead of traditional methods solving Euler-Lagrange equation. And Pereira et al. [16] proposed global and local fuzzy energy-based active contours (GL-FEAC) to deal with intensity images with inhomogeneity. Besides, the updating of average prototypes could be easily influenced by noise and outliers. Wu et al. [17] propose a novel region-based fuzzy active contour model with kernel metric by the minimization of a predefined energy function. A fuzzy global and local energy [18] is proposed and the local energy is constructed by considering both local spatial and gray level/color information.

In this paper, inspired by the FEAC model [15], we proposed a novel localized patch-energy active contour. In the present work, we make two main contributions.

1. By incorporating local patch-energy functional along each pixel of the evolving curve into the fuzziness of the energy, we construct a patch-based energy functional without regularization term. Its significant improvement is that objects which have heterogeneous statistics can be successfully segmented with localized fuzzy patch-based energies while corresponding global fuzzy region-based energies fail. In addition, the model not only avoids the periodical initialization during the evolution but also reduces the effect of noise.
2. To reject the minimal of the energy functional, we utilize a direct method to calculate the energy alterations instead of solving the Euler-Lagrange equation of the underlying problem.

## 2. Previous Work

Let *I*(*x*) : *Ω* → *R* be a given gray level image and *C* be a close contour. The piecewise constant energy functional in the Chan-Vese model [5] is defined by

(1) ECVC,c1,c2=λ1∫OutsideCIx−c12dx+λ2∫InsideCIx−c22dx+μ·lengthC,

where *λ*_1_, *λ*_2_, and *μ* are three positive constants and *c*_1_ and *c*_2_ are two constants that approximate the image intensity *I*(*x*) inside *C* and outside *C*. This energy function *E* ^CV^(*C*, *c*_1_, *c*_2_) can be represented by a level set formulation, and the energy minimization problem can be converted to solving a level set evolution equation.

To solve this minimization of the energy functional, the level set method [5] is represented by the unknown curve *C* as the zero level set of a Lipschitz function *ϕ*(*x*), such that

(2) ϕx>0if  x∈InsideC,ϕx=0if  x∈OnC,ϕx<0if  x∈OutsideC.

Thus, the energy functional *E* ^CV^(*c*_1_, *c*_2_, *C*) can be reformulated in terms of the level set function *ϕ*(*x*) as follows:

(3) EεCVc1,c2,ϕ=μ·∫Ωδεϕ∇ϕdx+λ1∫ΩIx−c12Hεϕdx+λ2∫ΩIx−c221−Hεϕdx,

where *H*_*ε*_ denotes the Heaviside function and *δ*_*ε*_(·) denotes the Dirac delta function defined as follows:

(4) Hεx=121+2πarctan⁡xε,δx=dHxdx=1π·εε2+x2,ε⟶0.

The Euler-Lagrange equations are used to solve this minimization problem in (3) and update the level set function by the gradient descent method. It is clear that the Chan-Vese model can deal with the detection of objects whose boundaries are either smooth or not necessarily defined by gradient. They do not require image smoothing and thus cannot efficiently process the images with noise. That is to say, the model is more sensitive to noise and cannot handle objects with ill-defined boundaries.

The FEAC model [15] combining the fuzzy sets with the active contour methodology aims to segment regions of interest into a two-phase image. It is provided to the algorithm with an initial partition of the image such as an initial contour. This partition defines the curve that will iteratively evolve by a minimization process. The evolving contour is implicitly represented as the pseudo zero level set of the LSF *u* such that we have the following definitions expressed by

(5) C=x∈I:ux=0.5,insideC=x∈I:ux>0.5,outsideC=x∈I:ux<0.5.

And the fuzzy energy region-based function is expressed by (5):

(6) FC,c1,c2,u=μ·LengthC+λ1∫ΩuxmIx−c12dx+λ2∫Ω1−uxmIx−c22dx,

where the parameters *μ* ≥ 0, *λ*_1_, *λ*_2_ > 0 are fixed parameters. The fuzzy membership functions *u*(*x*)∈[0,1] and (1 − *u*(*x*)) represent the membership value that pixel *x* belongs to *c*_1_ and *c*_2_, respectively. *m* is the weighting exponent on each fuzzy membership and usually set to 1 or 2. *c*_1_ and *c*_2_ are average prototypes of the original image inside *C* and outside *C*.

Keeping *u*(*x*) fixed and minimizing the energy function *F*(*C*, *c*_1_, *c*_2_, *u*) with respect to *c*_1_ and *c*_2_, it is easy to get the equations by updating the following values *c*_1_ and *c*_2_:

(7) c1=∫ΩuxmIxdx∫Ωuxmdx,c2=∫Ω1−uxmIxdx∫Ω1−uxmdx.

Furthermore, keeping *c*_1_ and *c*_2_ and fixed and minimizing the energy *F*(*C*, *c*_1_, *c*_2_, *u*) with respect to *u*, it is easy to express variable in the following way:

(8) $$\begin{array}{c} u\left(\;x\;\right)=\frac{1}{1+{\left(\lambda_{1}{\left(I\left(x\right)-c_{1}\right)}^{2}/\lambda_{2}{\left(I\left(x\right)-c_{2}\right)}^{2}\right)}^{1/\left(m-1\right)}}. \end{array}$$

Specifically, for a certain pixel *x*, we compute the fuzzy membership in this pixel using (8). Then, according to the change of Δ*F* caused by the single change of the fuzzy membership in pixel *x*_*i*_, we decide whether this new fuzzy membership replaces the old one in this pixel *x*_*i*_ or not. If the change of Δ*F* becomes negative, then the new fuzzy membership is adopted. If not, the old one is kept. However, by updating *u*, pixels in the background could be easily labeled as pixels belonging to the object region if their intensities are very close to the average prototype of the object region. Also, these approaches still need localized information to achieve reliable performance.

The remainder of this paper is organized as follows.  Section 3 describes the proposed model, including the model description, numerical approximation, and the description steps of the proposed model. Experiments and results, including experimental results and the scale parameter of the localization radius, are discussed in Section 4. Finally concluding remarks are given in Section 5.

## 3. The Proposed Model

### 3.1. The Model Description

**Figure 1:**
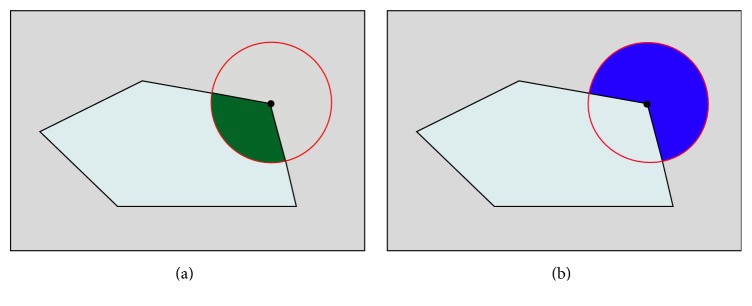
A circle patch partitioned into two parts is considered at each pixel (black dot in two images) along the evolution contour. (a) The bottle green region (object region) shows the interior region. (b) The blue region (background region) shows the exterior region.

Let the local patch (a circle region) *Ω*_*x*_ be centered at location *x*; the spatial variable *y* ∈ *Ω*_*x*_ is a single point and independent of spatial variable *x*. The local patch *Ω*_*x*_ with the radius *r* is represented as *Ω*_*x*_ = {|*y* − *x*| ≤ *r*, *y* ∈ *Ω*}. We define the mask function *W*(*x*, *y*) to describe the local patch *Ω*_*x*_ as described in Figure 1:

(9) $$\begin{array}{c} W\left(\;x,y\;\right)=\left\{\begin{array}{cc} 1, & y\in \Omega_{x},\\ 0, & otherwise. \end{array}\right. \end{array}$$

It is clear that the mask function *W*(*x*, *y*) will be 1 when the point *y* is within the local patch *Ω*_*x*_ and 0 otherwise. It is noticed that the value of radius *r* should be selected properly so as to capture enough local intensity information. In our work, we assume that the energies can be constructed of a family of localized patch-based energies at each point along the curves instead of global energies in the whole image. In order to formulate the local patch-based functional, we treat each point separately and split local neighborhoods into local interior and local exterior by the evolving curve.

In the model, the evolving contour is implicitly represented as the pseudo LSF based on the membership function *u* in (5) similar to the FEAC model [15]. For a given pixel *x* of image domain along the curves, the local patch-based functional by incorporating the fuzzy set is given as follows:

(10) Fx∫ΩuymIy−c12dx+∫Ω1−uymIy−c22dx=∫ΩyWx,y·uymIy−c12dx+∫ΩyWx,y·1−uymIy−c22dy,

where two constants *c*_1_ and *c*_2_ represent the intensity averages of *u*(*y*) > 0.5 and *u*(*y*) < 0.5. The local patch-based energy function *F*_*x*_ is an internal measure functional to express the local adherence in the proposed model at each point along the evolving contour. The pseudo LSF *u*(*x*) represent the degree of the pixel *I*(*y*) belonging to the interior region. The overall localized patch-based functional can be formulated as follows:

(11) FC,c1,c2,u=∫ΩxFx dx=∫Ωx∫ΩyWx,y·uymIy−c12dy dx+∫Ωx∫ΩyWx,y·1−uymIy−c22dy dx.

To compute the energy functional *F*, we ignore the contributions from the points far away from the current computed point since computing a big number of the points in the whole image requires more computation. To decrease the impact caused by inhomogeneity that may arise far away, we widen a range of objects because narrow range only captures limited objects. Note that we do not consider the regularization term of the energy functional. For any point *x* in the local patch, we use the mask function *W*(*x*, *y*) to make sure that only on local patch information centered at *y* can be computed. Thus, the contribution of the total energy functional is the sum of all neighborhood points along the evolving contour. In the following, we will use two steps to solve functional (11) in the following.

**Step 1 .**

Keeping *u*(*y*) fixed and computing the minimization of functional (11) with regard to *c*_1_ and *c*_2_, we can easily get these constant functions by

(12) c1=∫ΩyWx,y·uymIydy∫ΩyWx,y·uymdy,c2=∫ΩyWx,y·1−uymIydy∫ΩyWx,y·1−uymdy.

**Step 2 .**

Keeping the variables *c*_1_ and *c*_2_ fixed and computing the minimization of energy (11) with regard to *u*(*y*), we can get the following Euler-Lagrange equation:

(13) ∫ΩxWx,y·uym−1Iy−c12dx−∫ΩxWx,y·1−uym−1Iy−c22dx=0.

From the above equation, the variable *u*(*y*) can be expressed in the following way:

(14) $$\begin{array}{c} u\left(\;y\;\right)=\frac{1}{\left(1+{\left({\left(I\left(y\right)-c_{1}\right)}^{2}/{\left(I\left(y\right)-c_{2}\right)}^{2}\right)}^{1/\left(m-1\right)}\right)}. \end{array}$$

The variable *u*(*y*) is then updated based on the change of the energy *F*.

### 3.2. Numerical Approximation

Since the energy functional (11) is nonconvex, it is difficult to solve the minimization in practice. Generally, the gradient descent schema driven by the Euler-Lagrange equation is usually applied to explicit time marching and causes local minimal. To solve this problem, inspired by the scheme developed by Krinidis and Chatzis [15] and Pereira et al. [16], we apply a fast numerical scheme to make its time step unconstrained in the explicit time marching. The algorithm can calculate the energy directly and judge if the degree of membership for any point is changed instead of solving the partial differential equation.

**Lemma 1 .**

Let *P* ∈ *I* be a given point in the local patch *Ω*_*y*_, the intensity value of point *P* be *I*_0_, and the corresponding degree of membership for this point *P* be *u*_0_. Correspondingly, let the new value of the degree of membership at the same point *P* be *u*_*n*_; the values of *c*_1_ and *c*_2_ will be changed to new two ones: ${\hat{c}}_{1}$ and ${\hat{c}}_{2}$. The new value of ${\hat{c}}_{1}$ and ${\hat{c}}_{2}$ could be calculated as follows:

(15) c^1=c1+unm−u0ms1+unm−u0mI0−c1,c^2=c2+1−unm−1−u0ms2+1−unm−1−u0mI0−c2,

where *s*_1_ = ∑_*Ω*_*y*__ *W*(*x*, *y*) · [*u*(*y*)]^*m*^ and *s*_2_ = ∑_*Ω*_*y*__ *W*(*x*, *y*) · [1 − *u*(*y*)]^*m*^.

**Proof**

The two old constants *c*_1_ and *c*_2_, which approximate the image intensity in local patch *O*_*y*_ corresponding to *u*(*y*) > 0.5 and *u*(*y*) < 0.5 in (12), respectively, are written in the following forms:

(16) c1=∑ΩyWx,y·uymIydy∑ΩyWx,y·uymdy,c2=∫ΩyWx,y·1−uymIydy∫ΩyWx,y·1−uymdy.

Assuming that we change the degree of membership for only one point *P* when we compute the new degree of membership *u*_*n*_ for the point *P*, the constant ${\hat{c}}_{1}$ will be obtained by

(17) c^1∑ΩyWx,y·u^ymIy∑ΩyWx,y·u^ym=∑ΩyWx,y·uymIy+unmI0−u0mI0∑ΩyWx,y·uym+unm−u0m=s1c1+I0unm−u0ms1+unm−u0m=c1+unm−u0ms1+unm−u0mI0−c1,

where *s*_1_ = ∑_*Ω*_*y*__ *W*(*x*, *y*) · [*u*(*y*)]^*m*^.

In a similar way, we obtain the new value ${\hat{c}}_{2}$ as

(18) $$\begin{array}{c} {\hat{c}}_{2}=c_{2}+\frac{{\left(1-u_{n}\right)}^{m}-{\left(1-u_{0}\right)}^{m}}{s_{2}+\left({\left(1-u_{n}\right)}^{m}-{\left(1-u_{0}\right)}^{m}\right)}\left(\;I_{0}-c_{2}\;\right), \end{array}$$

where *s*_2_ = ∑_*Ω*_*y*__ *W*(*x*, *y*) · [1 − *u*(*y*)]^*m*^.

Thus, the changed values $\Delta c_{1}={\hat{c}}_{1}-c_{1}$ and $\Delta c_{2}={\hat{c}}_{2}-c_{2}$ for the point *P* can be very easily computed using formulations (17) and (18), respectively. This completes the proof.

**Lemma 2 .**

Let the old total energy functional be *F* and the new total energy functional be $\hat{F}$ when we change the degree of membership for the point *P* into *u*_*n*_. Correspondingly, the changed energy Δ*F* between the new and old total energy functional is given as follows:

(19) ΔF=∑Ωxs1unm−u0ms1+unm−u0mI0−c12+s21−unm−1−u0ms2+1−unm−1−u0mI0−c22.

**Proof**

For the fixed point *P* in the model, the change of the degree membership will lead to the change of the new energy functional. To compute the alteration $\Delta F=\hat{F}-F$ between the new and old total energy functional, we firstly calculate the new energy functional $\hat{F}$, which is written in the following form:

(20) F^=∑Ωx∑ΩyWx,y·u~ymIy−c~12︷A1︸A~+∑Ωx∑ΩyWx,y·1−u~ymIy−c~22︷B1︸B~.

From above, we can see that we should separately computer $\tilde{A}$ and $\tilde{B}$ in order to calculate the alteration. So

(21) A~=∑Ωx∑ΩyWx,y·u~ymIy−c~12=∑Ωx∑ΩyWx,y·uymIy−c~12+unm−u0mI0−c~12.

We will first compute the following equations $(I(y)-{\tilde{c}}_{1}{)}^{2}$ and $(I_{0}-{\tilde{c}}_{1}{)}^{2}$:

(22) Iy−c~12=Iy−c1−unm−u0ms1+unm−u0mI0−c12=Iy−c12−2Iy−c1unm−u0ms1+unm−u0mI0−c1+unm−u0ms1+unm−u0mI0−c12,I0−c~12=I0−c1−unm−u0ms1+unm−u0mI0−c12=s1I0−c1s1+unm−u0m2.

Then we will insert (22) into (20), we have the following equation ${\tilde{A}}_{1}$:

(23) A~1=A1+unm−u0ms1+unm−u0mI0−c12·∑ΩyWx,y·uym−2unm−u0ms1+unm−u0mI0−c1·∑ΩyWx,y·uymIy−c1+unm−u0m·s1I0−c1s1+unm−u0m2=A1+unm−u0ms1+unm−u0mI0−c12s1−2·unm−u0ms1+unm−u0mI0−c1s1Iy−s1c1+unm−u0ms1I0−c1s1+unm−u0m2=A1+unm−u0mI0−c12·s1unm−u0ms1+unm−u0m2+s12s12+unm−u0m2=A1+s1unm−u0ms1+unm−u0mI0−c12.

For the image domain *Ω*_*x*_, we have

(24) A~∑ΩxA~1=∑ΩxA1+s1unm−u0ms1+unm−u0mI0−c12=A+∑Ωxs1unm−u0ms1+unm−u0mI0−c12,

where *A*_1_ = ∑_*Ω*_*y*__ *W*(*x*, *y*) · *u*(*y*)^*m*^(*I*(*y*) − *c*_1_)^2^ and *A* = ∑_*Ω*_*x*__∑_*Ω*_*y*__ *W*(*x*, *y*) · *u*(*y*)^*m*^(*I*(*y*) − *c*_1_)^2^.

In the same way, we can get $\tilde{B}$

(25) B~=∑ΩxB~1=B+∑Ωxs2·1−unm−1−u0ms1+1−unm−1−u0mI0−c22,

where *B* = ∑_*Ω*_*x*__∑_*Ω*_*y*__ *W*(*x*, *y*)·(1 − *u*(*y*))^*m*^(*I*(*y*) − *c*_2_)^2^.

Combining (20), (24), and (25), the new total energy functional is rewritten as

(26) F^=F+∑Ωxs1unm−u0ms1+unm−u0mI0−c12+s21−unm−1−u0ms1+1−unm−1−u0mI0−c22.

So $\Delta F=\hat{F}-F=\sum_{\Omega_{x}}(s_{1}\left(\left(u_{n}^{m}-u_{0}^{m}\right)/\left(s_{1}+u_{n}^{m}-u_{0}^{m}\right)\right){\left(I_{0}-c_{1}\right)}^{2}$ + *s*_2_(((1 − *u*_*n*_)^*m*^ − (1 − *u*_0_)^*m*^)/(*s*_1_ + (1 − *u*_*n*_)^*m*^ − (1 − *u*_0_)^*m*^))(*I*_0_ − *c*_2_)^2^), the end of proof.

Thus, it is very easy to compute the changes $\Delta F=\hat{F}-F$ by updating *c*_1_ and *c*_2_, when the change at the degree of membership of a point *P* is occurred.

**Remark 3 .**

It is required that local region statistics must be calculated for all the points along the evolving curve. So it increases the complexity of the algorithm and the computation time. To improve the computational efficiency, we only update the membership *u*(*y*) in a narrow band region around the pseudo level set function *u*(*y*) = 0.5. In this paper, the computation of local statistics is separated into two parts. In the first part, the local region-based method begins by initializing every pixel in the narrow band with the local interior and exterior statistics. In the other part, the statistical models of all pixels within the narrow band neighborhoods are updated when any initialized pixel is crossed by the contour moving it from the interior to the exterior or vice versa.

### 3.3. Description of Algorithm Steps

Here, the segmentation procedure of the proposed model is summarized as follows.

**Step 1 .**

Give an initial partition of the image, set *u*_0_ > 0.5 for one part and *u*_0_ < 0.5 for the other.

**Step 2 .**

Compute the values *c*_1_, *c*_2_, and *u*_0_ for the whole image *I* using (17) and (18).

**Step 3 .**

Assume that the intensity value of point *P* be *I*_0_ and the corresponding degree of membership for the point *P* be *u*_0_. Calculate the new degree of membership *u*_*n*_ using (14) for current pixel *I*_0_ and the difference between the new and old energy Δ*F* using (26).

If Δ*F* ≥ 0, then change *u*_0_ with *u*_*n*_ value; otherwise, keep the old value *u*_0_.

**Step 4 .**

Repeat Step 3 to compute the total energy *F* within the narrow band neighborhood using Jacobi iterations. When all pixels within the neighborhood image have been swept one time, one iteration is finished. The updated values of the current iteration are used for the next iteration.

**Step 5 .**

Repeat Steps 2–4 till the total energy *F* remains unchanged.

## 4. Experiments and Results

In the section, we will validate that localized patch-based model can improve the performance of a given global energy instead of specially comparing with the fuzzy region-based global energies. To demonstrate the performance of the proposed model, we also test the segmentation results using the proposed model, the FEAC model [15], and the GL-FEAC model [16] on different synthetic images and real images. The experiment results will demonstrate that only the localized model can obtain a correct segmentation in these cases. All experiments are performed on a 1.86-GHz Intel dual-core notebook computer with Memory 3 GB using the MATLAB programming language. In these experiments, the parameter *m* is 2. The code will be uploaded to the website: http://fangjx2005.com/.

### 4.1. Experiment Results

**Figure 2:**
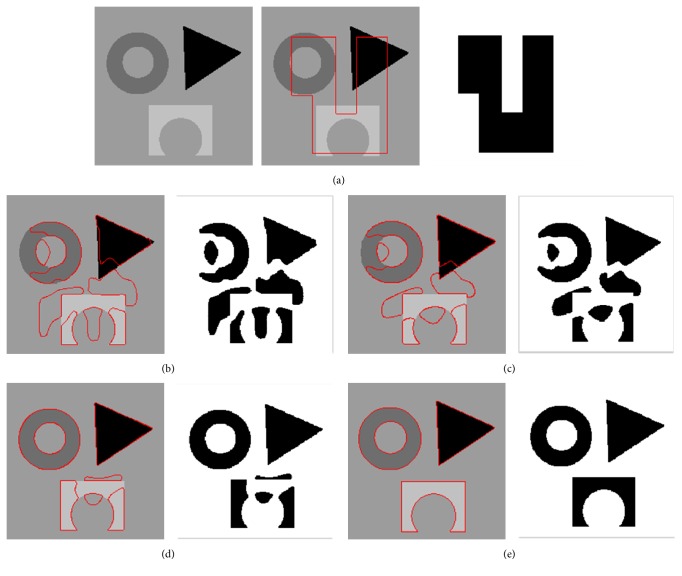
Segmentation results on a synthetic image with three object regions using the proposed model. The curve evolution processes from the initial contour to the final contours are shown. (a) The original image, the original curve image, and the initial contour. (b, c, d, e) Three intermediate results and final contours with the object regions.

**Figure 3:**
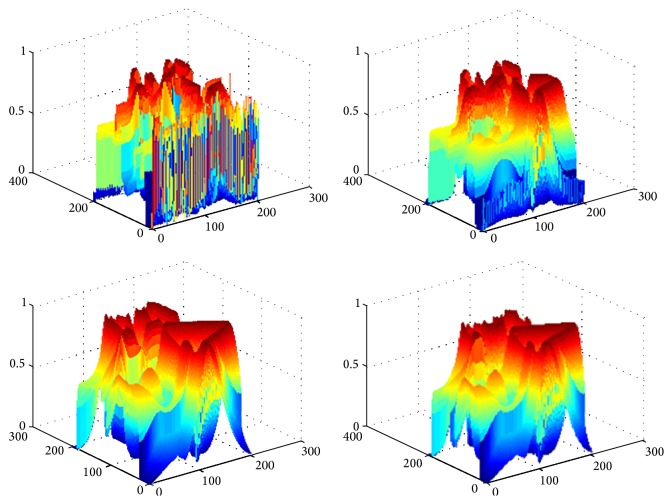
The evolution of the contours (the pseudo level set function) corresponding to the three intermediate contours and final contour in Figure 2, respectively.

Figure 2 shows that the proposed model segments a synthetic image with three different objects. In the experiment, the parameter of the localization radius is set to 20. For each image region, the image includes different intensities and holes in the interior of the objects.  Figure 2(a) shows the original image, the original curve image, and the initial contour, respectively. To better describe the curve evolution, three intermediate results with object regions are shown in Figures 2(b)–2(d).  Figure 2(e) depicts the final segmentation results using the proposed model. Four pseudo level set functions corresponding to three intermediate evolution contours and final contours are shown in Figure 3. It can be seen from these results that the proposed model is able to extract all object regions though the desired objects include different intensities.

**Figure 4:**
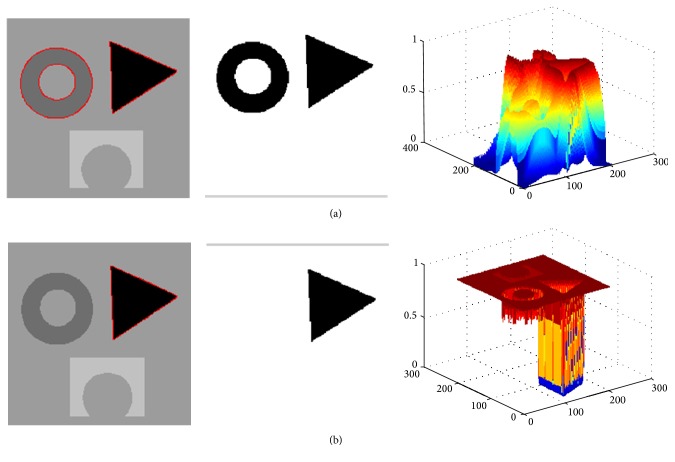
Segmentation results on the same synthetic image using the global FEAC model. (a) The intermediate curve position, the segmentation image, and the evolution of the contours. (b) The final curve position, the segmentation image, and the evolution of the contour.

In Figure 4, we applied the global FEAC model to segment the synthetic image with the same initial curve. The intermediate curve image, the segmentation result, and the evolution of the contours are shown in Figure 4(a).  Figure 4(b) shows the final curve position, the segmentation image, and the evolution of the contour after 200 iterations. The segmentation results show only one of three objects is segmented even if the synthetic image appears simple. It is obvious that the global energy functional in the FEAC model cannot extract the exact boundaries since it only finds the most distinct parts of the image. These examples demonstrate that the FEAC model causes significant problems for segmenting the image with slight intensity inhomogeneity.

**Figure 5:**
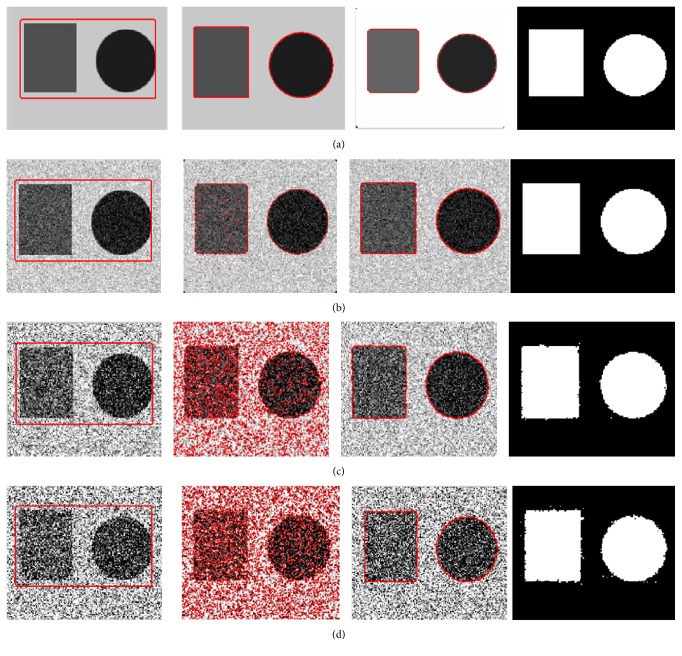
Segmentation results on synthetic image mixed with different Gaussian noises using the FEAC model and the proposed model. (a) The clean image. (b) Gaussian noise with variance 0.01. (c) Gaussian noise with variance 0.10. (d) Gaussian noise with variance 0.2. From left to right: initial contour, the segmentation results using the FEAC model, the segmentation results using the proposed model, and the final object regions using the proposed model.

The next experiment is to test the robustness to noise of the proposed model and the results are shown in Figure 5. It is carried out on synthetic images mixed with different Gaussian noises using the FEAC model and the proposed model. The objects in the image include two kinds of shapes (*circle* and* rectangle*). From left to right, Figure 5 shows initial contour, the segmentation results using the FEAC model, the segmentation results using the proposed model, and the final object regions using the proposed model, respectively. From Figures 5(b)–5(d), it shows the segmentation results on conditions that the images are mixed with Gaussian noise with variances 0.01, 0.10, and 0.20, respectively. The results show that more noise causes too more small segmentation regions using the FEAC model. From these segmentation results, the proposed model can accurately extract the boundary while the FEAC model cannot get satisfactory result even if the image is populated by severe noise. Thus, we can see that our method shows the better robustness to noise.

**Figure 6:**
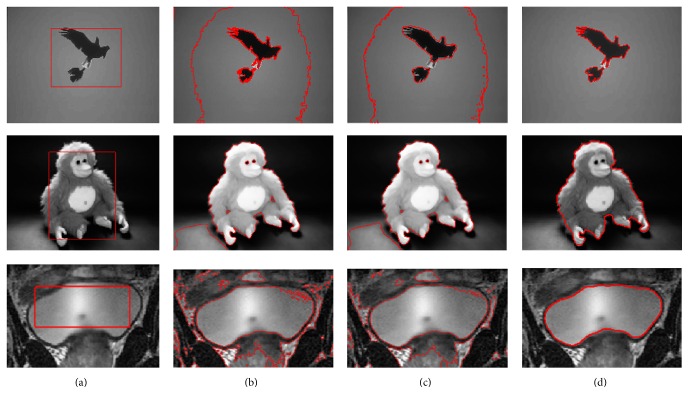
Segmentation results for the BIRD, MONKEY, and medical images using different methods. (a) shows the initialization; (b), (c), and (d) show the segmentation results using the FEAC model, GL-FEAC model, and the proposed model, respectively.

In Figure 6, we further compare the proposed model with these global fuzzy active contour models, such as the FEAC model and the GL-FEAC model, on BIRD, MONKEY, and medical images. In the GL-FEAC model, the parameters are set as follows: *β* = 0.5, *λ*_1_ = *λ*_2_ = 1. The images in Figure 6 show objects and backgrounds which are multimodal but that have intensities that change smoothly and quickly.  Figure 6(a) shows the initial curve image. In Figures 6(b), 6(c), and 6(d), the boundary is obtained using the FEAC model, the GL-FEAC model, and the proposed model, respectively. From the segmentation results, we can see that the global energy finds only the brightest parts of the image while the localization stops on object boundaries.

In this paper, we use the popular error metric, the Dice coefficient [19], to quantitatively evaluate the performance of the competing methods. The Dice coefficient between two regions *A* and *B* is calculated as *D*(*A*, *B*) = 2×|*A*∩*B* | /(|*A* | +|*B*|), where |*A*∩*B*|, |*A*|, and |*B*| denote the pixel number of their union areas *A* and *B* and *A* and *B*, respectively. Obviously, the closer the Dice coefficient value is to 1, the better the segmentation results we will get.  Table 1 depicts the Dice coefficient which gives the quantitative comparison scores in Figure 6.

**Figure 7:**
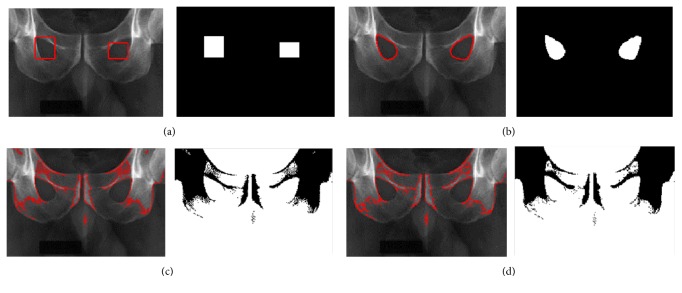
Segmentation results on PELVIS image using different models. (a) shows the initialization; (b), (c), and (d) show the segmentation results using the proposed model, the FEAC model, and the GL-FEAC model, respectively.

We next illustrate the advantage of the proposed contour model on the PELVIS image in order to extract two object regions (the left obturator foramen and right obturator foramen) and the results are shown in Figure 7. The localization radius is 20. The PELVIS image with intensity inhomogeneity has blur boundary in some parts. The segmentation renders a challenging task for extracting the desired object boundaries.  Figure 7(a) shows the initial contour image with two contours (two rectangles). The segmentation results in Figures 7(b)–7(d) are obtained using the proposed model, the FEAC model, and the GL-FEAC model, respectively. From these results, the global energies in the FEAC and GL-FEAC models can only segment the brightest regions of the whole image, whereas our localization model can extract two desired objects and get the accurate results.

**Figure 8:**
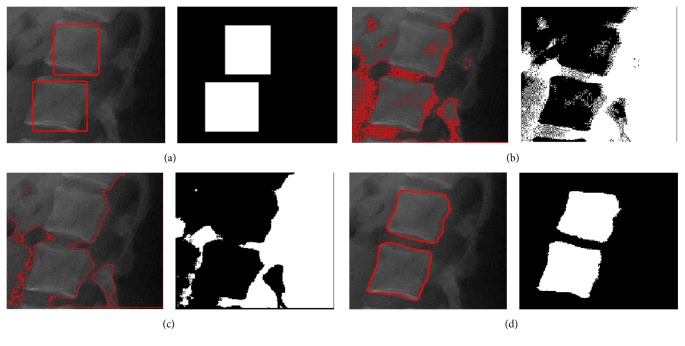
Segmentation results on lumbar spine image with two object regions using different models. (a) shows the initialization; (b), (c), and (d) show the segmentation results using the proposed model, the FEAC model, and the GL-FEAC model, respectively.

**Figure 9:**
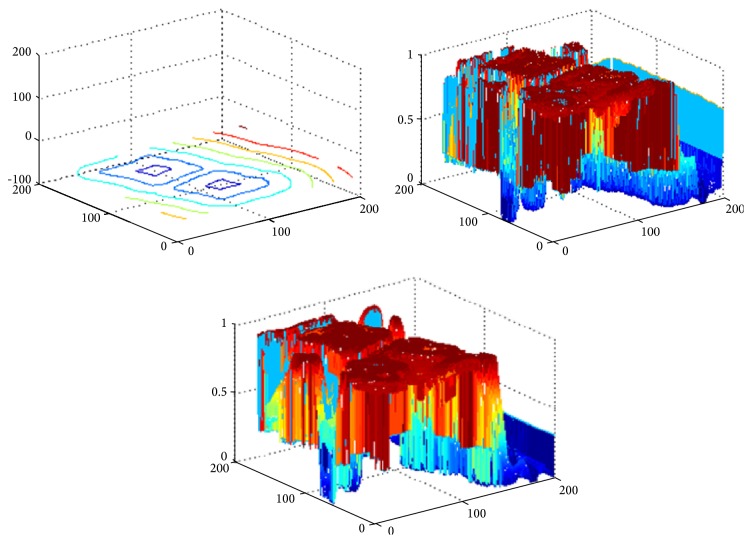
The evolution of the contours corresponding to the proposed model, the FEAC model, and the GL-FEAC model in Figure 8, respectively.

We performed an additional experiment to show the effect of the proposed model on a lumbar spine image. Compared with the PELVIS image shown in Figure 7, the image has more seriously blurred boundary and is much more complicated.  Figure 8 shows the segmentation results using the FEAC model, the GL-FEAC model, and the proposed model with the same local radii.  Figure 8(a) shows the initial contour image with two contours (two rectangles). In Figures 8(b) and 8(c), these global models can only extract the brightest regions and cannot extract the exact boundaries. With the proposed model and the same initial position, the boundaries of two lumbar spine regions are successfully recovered as depicted in Figure 8(d). It is clear that only the proposed model could only extract the object boundaries. The evolution of the contours corresponding to the FEAC model, the GL-FEAC model, and the proposed model is shown in Figure 9.  Table 2 reports the Dice measure for segmentation results by the FEAC model, GL-FEAC model, and the proposed model in Figures 7 and 8.

**Figure 10:**
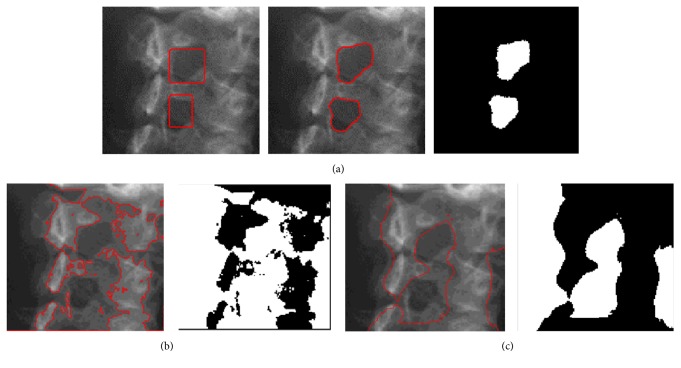
Comparison of different methods for cervical spine image segmentation. In (a), the segmentation result and the object image using the proposed model are shown. (b, c) show the segmentation results using the* FAC-Ker* and* GLFAC* models, respectively.

In the following experiment, Figure 10 shows different segmentation results for cervical spine images with noise. The typical schemes include the fuzzy active contour model with kernel metric (*FAC*-*Ker*) [17] and the global and local fuzzy active contour with the information (*GLFAC*) [18]. These models have the same initial positions. The initial contour image, the segmentation result, and the object image using the proposed model are shown in Figure 10(a). The results indicate that only the proposed model can extract two objects. In Figures 10(b) and 10(c), it shows the segmentation results using the* FAC*-*Ker* and* GLFAC* models, respectively. From the segmentation results, we can see that the* FAC*-*Ker* model can lead to poor separation of different regions because the model is based on global image information. The* GLFAC* model can reduce the noise, but it cannot extract the objects.

### 4.2. The Scale Parameter: Localization Radius

The important parameter in our model is localization radius which plays a key role in how local object region(s) the proposed model will extract. As such, it should be selected based on the scale of the extracted object of interest and approximation of the surrounding region of clutter. A small localization radius is selected when we attempt to extract small objects with nearby clutter and vice versa.

**Figure 11:**
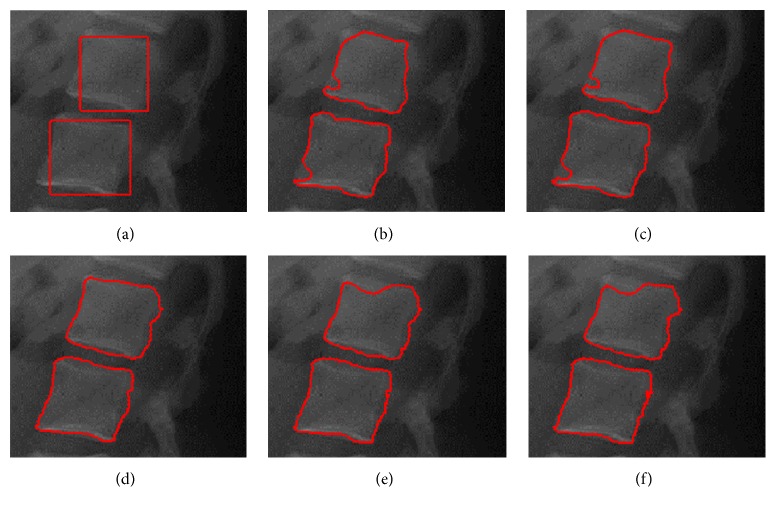
Segmentation results using the proposed method with different localization radii. (a) shows the initialization on lumbar spine image; (b)–(f) show the resulting segmentation with means separation energy using localizing radius 10, 15, 20, 25, and 30, respectively.

The example of lumbar spine image segmentation in Figure 11 illustrates the effect of different localization radii. In all these experiments, the number of the iterations is 200. The segmentation results are shown in Figures 11(b)–11(f) using the proposed model with localization radius 10, 15, 20, 25, and 30, respectively. With the same initialization, Figure 11 shows different results using five different local radii. From the segmentation results in Figures 11(b) and 11(c), the smallest radius size results in an incorrect segmentation that is too local, whereas the largest radius in Figures 11(e) and 11(f) leads to an incorrect energy value that is too global for the task at hand. Thus, the localization radius should be correctly chosen in terms of the nature of the objects so that the neighborhood is large enough to detect the desired boundary from the initialization.  Table 3 describes the Dice measure for segmentation results with different localization radius.

## 5. Conclusion

In this paper, we propose a localized patch-energy fuzzy active contour model by incorporating local image statistics for each pixel into the fuzziness of the energy, which can avoid local minima of the energy functional. In addition, we use a fast numerical method directly computing the changed value of the energy functional to update the curve evolution. Experiments show that the evolution of contour in our model is more stable which results in not only faster computation efficiency but also better performance of segmentation. Moreover, the alterations of the energy in the energy functional are calculated directly instead of solving the Euler-Lagrange equation. Thus, the active contour converges quickly to the object boundaries. Experimental results on synthetic and real images have validated the effectiveness of the proposed model.

## Figures and Tables

**Table 1:** Average Dice coefficients values for different schemes on three different images.

The name of images	Size (pixel)	FEAC	GL-FEAC	Our
Bird	400 × 320	0.184274	0.276782	**0.947548**
Monkey	320 × 240	0.421280	0.491875	**0.894213**
Medical image	180 × 107	0.2547548	0.283295	**0.878747**

**Table 2:** Average Dice coefficients values for different schemes.

The name of image	Size	FEAC	GL-FEAC	Our
Pelvis image in Figure 7	216 × 151	0.164674	0.196280	**0.916548**
Lumbar spine image in Figure 8	199 × 171	0.128035	0.184621	**0.854213**

**Table 3:** Average Dice coefficients values for localizing radius.

The localizing radius	Size: 10	Size: 15	Size: 20	Size: 25	Size: 30
Average Dice coefficients	0.586413	0.676421	0.854213	0.764125	0.685462

## References

[1] Osher S., Sethian J. A. (1988). Fronts propagating with curvature-dependent speed: algorithms based on Hamilton-Jacobi formulations. *Journal of Computational Physics*.

[2] Kass M., Witkin A., Terzopoulos D. (1988). Snakes: active contour models. *International Journal of Computer Vision*.

[3] Caselles V., Kimmel R., Sapiro G. (1997). Geodesic active contours. *International Journal of Computer Vision*.

[4] Mumford D., Shah J. (1989). Optimal approximations by piecewise smooth functions and associated variational problems. *Communications on Pure and Applied Mathematics*.

[5] Chan T. F., Vese L. A. (2001). Active contours without edges. *IEEE Transactions on Image Processing*.

[6] Zhu S. C., Yuille A. (1996). Region competition: unifying snakes, region growing, and Bayes/MDL for multiband image segmentation. *IEEE Transactions on Pattern Analysis and Machine Intelligence*.

[7] Fang J., Yang J., Tu E., Jia Z., Kasabov N. K. (2011). Efficient multiresolution level set image segmentation with multiple regions. *Optical Engineering*.

[8] Fang J., Yang J., Tu E. M., Jia Z., Kasabov N., Liu C. (2011). Statistical approaches to automatic level set image segmentation with multiple regions. *Optical Engineering*.

[9] Ayed I. B., Mitiche A., Belhadj Z. (2005). Multiregion level-set partitioning of synthetic aperture radar images. *IEEE Transactions on Pattern Analysis and Machine Intelligence*.

[10] Vese L. A., Chan T. F. (2002). A multiphase level set framework for image segmentation using the Mumford and Shah model. *International Journal of Computer Vision*.

[11] Li C., Kao C.-Y., Gore J. C., Ding Z. (2008). Minimization of region-scalable fitting energy for image segmentation. *IEEE Transactions on Image Processing*.

[12] Li C., Kao C.-Y., Gore J. C., Ding Z. Implicit active contours driven by local binary fitting energy.

[13] Brox T., Cremers D. (2009). On local region models and a statistical interpretation of the piecewise smooth Mumford-Shah functional. *International Journal of Computer Vision*.

[14] Lankton S., Tannenbaum A. (2008). Localizing region-based active contours. *IEEE Transactions on Image Processing*.

[15] Krinidis S., Chatzis V. (2009). Fuzzy energy-based active contours. *IEEE Transactions on Image Processing*.

[16] Pereira C. L., Bastos C. A. C. M., Ren T. I., Cavalcanti G. D. C. Fuzzy active contour models.

[17] Wu Y., Ma W., Gong M., Li H., Jiao L. (2015). Novel fuzzy active contour model with kernel metric for image segmentation. *Applied Soft Computing*.

[18] Mondal A., Ghosh S., Ghosh A. (2016). Robust global and local fuzzy energy based active contour for image segmentation. *Applied Soft Computing*.

[19] Dice L. R. (1945). Measures of the amount of ecologic association between species. *Ecology*.

